# Antibiotic Treatment and Length of Hospital Stay in Relation to Delivery Mode and Prematurity

**DOI:** 10.1371/journal.pone.0164126

**Published:** 2016-10-07

**Authors:** Katia M. Ahlén, Anne K. Örtqvist, Tong Gong, Alva Wallas, Weimin Ye, Cecilia Lundholm, Catarina Almqvist

**Affiliations:** 1 Dept of Medical Epidemiology and Biostatistics, Karolinska Institutet, Stockholm, Sweden; 2 Institute of Environmental Medicine, Karolinska Institutet, Stockholm, Sweden; 3 Astrid Lindgren Children’s Hospital, Lung and Allergy Unit, Karolinska University Hospital, Stockholm, Sweden; University of Sydney, AUSTRALIA

## Abstract

**Aim:**

To investigate how 1) maternal delivery mode and 2) prematurity in infants are associated to antibiotic treatment and length of hospital stay.

**Methods:**

Women having given birth and infants 0–12 months discharged from hospital between July 2005 and November 2011 were identified from the Swedish National Patient Register. Medical records were reviewed for 203 women and 527 infants. The risk ratio (RR) between antibiotic treatment and 1) delivery mode in women; 2) prematurity in infants was calculated. Length of stay and days of antibiotic therapy were compared by Wilcoxon rank-sum test.

**Results:**

Women: There was an association between emergency caesarean section (CS) and antibiotic treatment (RR 5.0 95% confidence interval (CI) 2.2–11.5), but not for elective CS. Length of stay was longer for CS (emergency and elective) compared to vaginal delivery (p<0.01). Infants: RR for antibiotic treatment in preterm compared to term infants was 1.4 (95% CI 1.0–1.9). Length of stay (p<0.01), but not days of therapy (p = 0.17), was higher in preterm compared to term infants.

**Conclusion:**

We found that emergency CS increased the probability of maternal antibiotic treatment during hospitalisation, but no difference was found between term and preterm infants. The results are well aligned with current guidelines and may be considered in future studies on the effects of antibiotics.

## Introduction

Antibiotics are commonly used worldwide to treat bacterial infections in children and adults. In Sweden, one of three patients receive antibiotics during hospitalisation and hospitals account for 11% of the total national antibiotic use [1]. Information on dispensed antibiotics at an individual level in outpatient care is available through the Swedish Prescribed Drug register (SPDR) [2], but there is no equivalent register for inpatient care. Thus, information on the full individual exposure to antibiotics is currently unavailable. Although antibiotics are essential for treating serious bacterial infections, extensive use promotes antibiotic resistance and transmission of nosocomial infections [1]. Antibiotics given to the mother peri-partum may affect the pattern of infection in the child after birth [3] and early life exposure to antibiotics has been suggested to affect the colonisation of the gut flora and the developing immune system [3, 4]. Long term health consequences that have been linked to antibiotic exposure during fetal and early life include obesity [5, 6], allergic disease [7, 8] and asthma [8, 9]. However, further studies are needed to understand the causality and potential confounders affecting these associations [10, 11].

The average length of stay in Swedish hospitals for women giving birth was 2.2 days for vaginal delivery (VD) and 3.7 days for cesarean section (CS) in 2009 [12]. A survey of clinical guidelines in Swedish obstetric clinics showed that most clinics recommend antibiotic prophylaxis for emergency CS (which holds a higher risk of maternal post-partum infection), but not for elective CS [13]. Antibiotic prophylaxis is used during VD if risk factors for vertical transmission of Group B- Streptococci exist or in pre-labour premature rupture of membranes (pPROM) in week 22–32, to reduce risk of chorioamnionitis and neonatal infection [14]. Apart from the knowledge of current guidelines, we are not aware of any previous study that has investigated the relation between different delivery modes and the probability of antibiotic treatment in mothers giving birth

Bacterial infections in infants often present with unspecific symptoms and are difficult to diagnose, which may lead to both over-treatment and under-treatment with antibiotics [15]. Neonates with clinical symptoms of sepsis are commonly given empirical antibiotic treatment before confirmation of bacterial origin to prevent mortality in neonatal sepsis [16]. Preterm infants have a less developed innate immune system than term infants, with an increased risk for bacterial infections [17]. Furthermore, since maternal infection is one important mechanism contributing to spontaneous preterm births, preterm infants are more likely to be exposed to pathogens intra-uterine [18]. Preterm infants also have an increased length of stay compared to term infants [19] and are more often exposed to invasive medical devices during hospitalization [17], which increases the risk of nosocomial infections. Thus, it is likely that preterm infants are treated with antibiotics more frequently than term infants. Previous point prevalence studies have reported on general antibiotic use in inpatient care on a national level [20, 21] and we have recently reported proportions of antibiotics treatment with different childhood and maternal diagnoses [22], but comparisons between term and preterm birth have not been performed.

Since antibiotics given during the fetal and early life may affect the infant’s future health, it is of importance to study how pregnant women and infants are exposed to antibiotics in hospitals in addition to the available information from outpatient care. The aim of this study was to investigate the association between inpatient antibiotic treatment and delivery mode in pregnant women and prematurity in infants. Moreover, we aimed to examine how length of stay and days of therapy differed between the groups.

## Patients and methods

### Study design and population

This cross-sectional population-based study was based on linkage between registers held by the National Board of Health and Welfare (NBHW); SPDR, the National Patient Register (NPR) and the Medical Birth Register (MBR). The Swedish Personal Identity Number (PIN) is a unique identifier for every resident in Sweden and enables linkage of registers. SPDR contains information on all prescribed drugs in outpatient care since July 2005, classified according to the Anatomical Therapeutic Chemical classifications system (ATC). NPR contains national information on all primary and secondary diagnoses in inpatient care since 1987. MBR was established in 1973 and includes data on more than 98% of all births in Sweden.

In 2011, a linkage between the NPR and SPDR was performed at the NBHW and a cohort of all pregnant women and all children 0–5 years with a discharge date in the NPR between 1st July 2005 and 30th of November 2011 were identified as previously described (24). In this present study three groups were selected from the initial cohort for further study, according to patient’s age and diagnosis in NPR; 1) women with a diagnosis of VD (according to the International Classification of Disease, 10^th^ revision (ICD-10): O80), elective CS (O82.0) or emergency CS (O82.1); 2) infants aged 0–12 months at discharge with a primary or secondary diagnosis of prematurity (ICD-10: P07.2 or P07.3) and 3) infants aged 0–12 months without a diagnosis of prematurity (*i*.*e*. term infants). Pregnancy was defined as the period between the estimated date of conception and the infants’ birth date, based on information on gestational age fromMBR. Term infants with a primary diagnosis of malformations or factors influencing health status (ICD-10 codes Q or Z) were excluded to obtain a healthy reference group. A random sample of hospital visits from each group (n = 250 women, n = 640 infants) were drawn by the NBHW and the patient’s PIN’s were provided. In order to target patients who were given antibiotics only during the hospitalisation, individuals with a dispensed prescription of antibiotics in SPDR at the same day or day after the discharge date in NPR were excluded.

### Requested medical records

**Fig 1:**
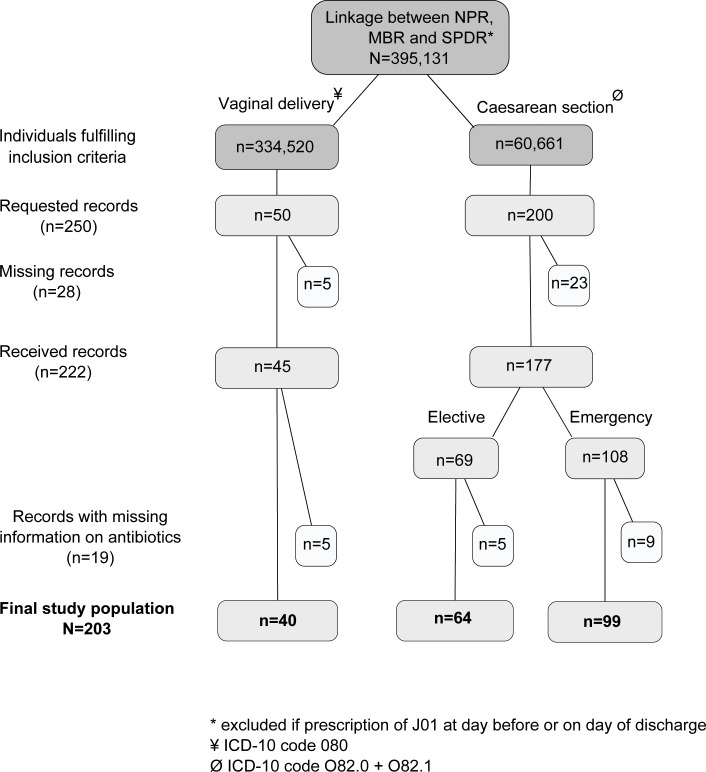
Study population and medical record request for pregnant women. Medical records were randomly selected from a register-based cohort of patients treated in Swedish hospitals between July 2005 and November 2011. Abbreviations: NPR = National Patient Register, MBR = Medical Birth Register, SPDR = Swedish Prescribed Drug Register, ICD-10 = International Classification of Disease 10^th^ revision.

**Fig 2:**
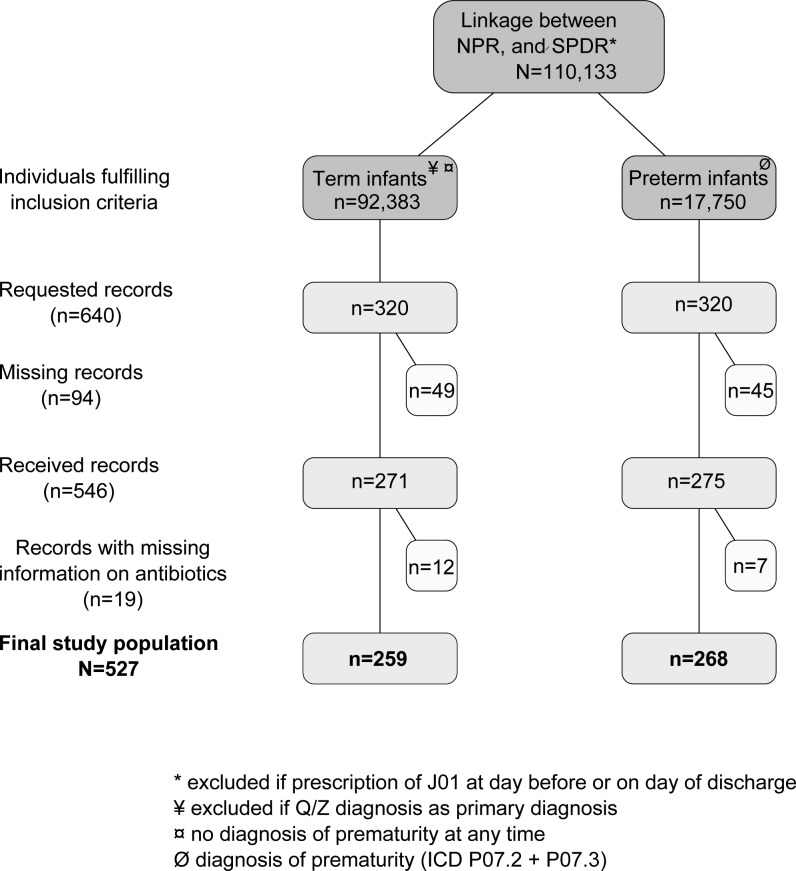
Study population and medical record request for infants 0–12 months. Medical records were randomly selected from a register-based cohort of patients treated in Swedish hospitals between July 2005 and November 2011. Abbreviations: NPR = National Patient Register, SPDR = Swedish Prescribed Drug Register, ICD-10 = International Classification of Disease 10^th^ revision.

Starting in January 2012, the study participants’ medical records, corresponding to the date of discharge and PIN, were requested by a letter to the head of the ward where the patient had been treated. Two reminder letters were sent out before the end of data collection in June 2013. Each medical record was manually reviewed and all available information regarding diagnosis, length of stay and potential antibiotic treatment was extracted. The numbers of requested and received medical records and the final study populations of women and infants are displayed in Figs 1 and 2. In total, medical records were received for 222 hospital visits for women and 546 hospital visits for infants, which correspond to a response rate of 89% and 85% respectively. Furthermore, thirty-eight hospital visits (19 women and 19 infants) were excluded due to missing information on antibiotic use, resulting in a final study population of 203 hospital visits for women and 527 hospital visits for infants.

### Variables

Information on women’s and infants’ age at admission and infants’ sex was extracted from the medical records. In women, age 30–34 years was chosen as reference group since it constituted the majority of individuals. Preterm infants were categorised as moderately preterm (born between 28–36 completed gestational weeks, ICD-10: P07.3) and extremely preterm (born before 28 completed gestational weeks, ICD-10: P07.2). Infant age was categorized as <3 months, 3–5, 6–8 and 9–12 months. Length of stay was defined as the period between date of admission and discharge. Administration of antibiotics was identified in the medical records by ATC-code J01. Antibiotic treatment was categorised as “yes”, if antibiotics were administered at any time during hospitalisation; “no” if antibiotics were not given. “Days of therapy” was defined as the total number of days with any or multiple types of antibiotic treatment. For each individual, treatment days for all administrations of antibiotics were summed. Individuals with missing information on both start- and end date, were excluded from the analysis on days of therapy. If information was missing only for the end date, treatment time for each administration was assumed to be one day. Information on start- and end date was complete only in half of the women treated with antibiotics. Thus, the analysis of days of therapy was only performed among infants.

### Statistical analysis

Risk ratio (RR) with 95% confidence intervals (CI) were estimated for antibiotic treatment in relation to delivery mode and degree of prematurity, according to Cornfield [23]. Since the age distribution of infants differed considerably between term and preterm infants, sub-analyses were performed for the age category with the majority of individuals (0–3 months). Descriptive statistics for length of stay (all individuals) and days of therapy (infants with antibiotic treatment in medical records) were presented in a box plot. Due to skewed distributions, two-sample Wilcoxon rank-sum test was used for comparisons between groups for length of stay and days of therapy. Cuzick’s non-parametric test for trend [24] was used to investigate the dose-response relationship for “term”, “moderately preterm”, and “extremely preterm” infants in relation to antibiotic treatment, length of stay and days of therapy. Significance was set at the 5% level. All analyses were performed using Stata statistical software, version 12.

### Ethical considerations

Consent to access the medical records was given by the head of each medical department. All data extracted from the medical records was de-identified prior to analysis and is presented in an aggregated way where no results may be connected to specific patients. The study has been approved by the Regional Ethical Review Board in Stockholm, Sweden.

## Results

### Pregnant women

**Fig 3:**
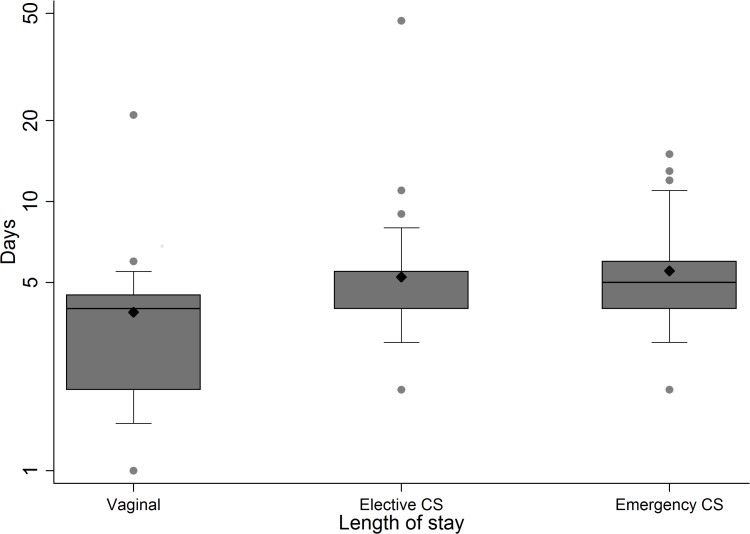
Boxplot with mean (◆) for length of stay for women by delivery mode. For elective CS, the median is equal to the lower quartile. Abbreviations: CS = Caesarean section

Table 1 displays antibiotic treatment in relation to delivery mode and age at delivery in pregnant women (N = 203). Almost two out of three women who gave birth with emergency CS (63%) were treated with antibiotics, while less than one in seven women who gave birth by elective CS (14%) or VD (13%) received antibiotics. Women delivering by emergency CS were thus five times more likely to receive antibiotics compared to women delivering by VD (RR 5.0, 95% CI 2.2–11.5), but no significant difference was found when comparing elective CS to VD. The proportion of women treated with antibiotics in relation to maternal age at delivery varied between 34% (25–29 years) and 47% (<25 years). However, the difference between age groups was not statistically significant. Length of stay was significantly higher for both emergency and elective CS compared to VD (p<0.01) (Fig 3).

**Table 1:** Risk ratios (RR) and 95% confidence intervals (CI) for antibiotic treatment in relation to delivery mode and maternal age at delivery (N = 203).

		All	Antibiotics		
		N	No, n (%)	Yes, n (%)	RR (95% CI)
**Delivery mode**	Vaginal	40	35 (87.5)	5 (12.5)	1 (ref)
	Elective CS	64	55 (85.9)	9 (14.1)	1.1 (0.4–3.1)
	Emergency CS	99	37 (37.4)	62 (62.6)	5.0 (2.2–11.5)
**Age (years)**	<25	17	9 (52.9)	8 (47.1)	1.3 (0.7–2.3)
	25–29	47	31 (66.0)	16 (34.0)	0.9 (0.6–1.5)
	30–34	74	47 (63.5)	27 (36.5)	1 (ref)
	>35	65	40 (61.5)	25 (38.5)	1.05 (0.7–1.6)

### Infants

The youngest age group (< 3 months) contained a majority of the infants in the study population (55% of the term and 92% of the preterm infants). The number of individuals in each of the age groups 3–5, 6–8 and 9–12 months decreased with increasing age. The majority of both term (61%) and preterm (59%) infants in the study population were males (data not tabulated). Table 2 presents antibiotic treatment in infants in relation to prematurity, sex and age at admission. In total, 22% of all infants were treated with antibiotics at least once during hospitalisation. RR for antibiotic treatment in preterm compared to term infants was 1.4, but the results did not reach statistical significance (95% CI 1.0–1.9). However, extremely preterm infants were twice as likely to receive antibiotics compared to term infants (RR 2.4, 95% CI 1.5–3.7) and there was a significant dose-response relationship for “term”, “moderately preterm”, and “extremely preterm” infants in relation to antibiotic treatment (p<0.01). There was no statistically significant association between antibiotic treatment and sex or age among term or preterm infants. Sub-analyses of antibiotic treatment in infants 0–3 months showed similar results as in the entire study population, with a small but not statistically significant difference between term and preterm infants (RR 1.2, 95% CI 0.8–1.8) and a doubled probability of antibiotic treatment in extremely preterm infants (RR 2.6, 95% CI 1.6–4.2) (S1 Table).

**Table 2:** Risk ratios (RR) and 95% confidence intervals (CI) for antibiotic treatment by prematurity, sex and age in infants 0–12 months of age.

		All	Antibiotics		
		N	No, n (%)	Yes, n (%)	RR (95% CI)
**All**		**527**	**409 (77.6)**	**118 (22.4)**	**-**
**Prematurity**	Term	259	210 (81.1)	49 (18.9)	1 (ref)
	Preterm	268	199 (74.2)	69 (25.8)	1.4 (1.0–1.9)
	*Moderately*	230	178 (77.4)	52 (22.6)	1.2 (0.8–1.7)
	*Extremely*	38	21 (55.3)	17 (44.7)	2.4 (1.5–3.7)
**Sex**	Male	317	251 (79.2)	66 (20.8)	1 (ref)
	Female	210	158 (75.2)	52 (24.8)	1.2 (0.9–1.6)
**Age (months)**	<3	389	295 (75.8)	94 (24.2)	1 (ref)
	3–5	64	53 (82.8)	11 (17.2)	0.7 (0.4–1.3)
	6–8	42	35 (83.3)	7 (16.7)	0.7 (0.3–1.4)
	9–12	32	26 (81.2)	6 (18.8)	0.8 (0.4–1.6)

**Fig 4:**
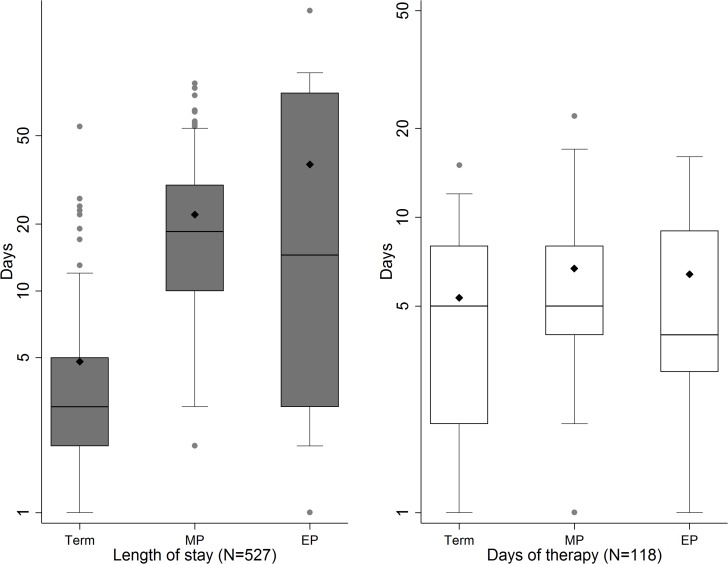
Boxplot with mean (◆) for length of stay and days of therapy for infants by degree of prematurity. Abbreviations: MP = Moderately premature, EP = Extremely premature

Fig 4 displays the distribution of length of stay and days of therapy for term, moderately preterm and extremely preterm infants. Length of stay was significantly higher in both moderately and extremely preterm infants compared to term infants (p<0.01), and showed a significant dose-response relationship (p<0.01). To calculate days of therapy for infants who had received antibiotics (n = 118), individuals with missing information on start- and end date were excluded (n = 9). Analysis was performed for the remaining 109 individuals and included 20 individuals (18.3%) where end date was missing and treatment time had been assumed to be one day. The median for days of therapy was 5 days for both preterm and term infants. No difference in days of therapy was found between term and preterm infants, neither when treating prematurity as dichotomous (Wilcoxon rank sum test, p = 0.17) or with degree of prematurity (Cuzick’s trend test, p = 0.30).

## Discussion

We found that women who delivered by emergency CS were five times more likely to receive antibiotics compared to women who delivered by VD or elective CS. Length of stay was significantly higher in women who delivered by CS compared to VD. Although antibiotic treatment in preterm infants overall did not reach statistical significance compared to term infants, extremely preterm infants were twice as likely to receive treatment as term infants. Length of stay was significantly higher in preterm infants compared to term infants, but no difference could be seen in days of therapy.

The prevalence of maternal antibiotic administration peri-partum varies globally. Global surveys performed in non-European countries by the World Health Organisation reported that antibiotics were administered to 47% of VD [25] and 87% of elective and emergency CS [26]. A Danish cohort study of 750 women found that antibiotics were given to 13% of VD and that antibiotic prophylaxis was given to all CS [27]. Several countries, such as Canada [28] and the United Kingdom [29], have implemented national guidelines recommending antibiotic prophylaxis for both elective and emergency CS. In contrast, our results from Swedish medical records suggest that antibiotics are commonly used in emergency CS but not as often in elective CS. This is in concordance with the local clinical guidelines from the Swedish Strategic Program against Antimicrobial Resistance [14] and a national survey of clinical guidelines [13].

Approximately 22% of the infants in this study received antibiotics during hospitalisation. Previous point prevalence studies by the Swedish Strategic Program against Antimicrobial Resistance have reported that an average of 29% of children aged 0–16 years were treated with antibiotics during hospitalisation [21]. Since we did not include patients who had a dispensed prescription of antibiotics in SPDR at the same day or day after the discharge, it is reasonable that we report a lower proportion of antibiotic treatment compared to the point prevalence study.

A large part of our study population consisted of neonates. Although neonatal sepsis is a relatively uncommon condition (3-4/1000 born children), it can develop rapidly and has a mortality of 9–15% [30]. Neonates with clinical symptoms of sepsis are routinely given empirical antibiotic treatment, before confirmation of bacterial origin, in order to prevent mortality. [16]. Early onset sepsis occurs within 72 hours after birth and the infection is often transmitted vertically by bacteria in the mother’s urogenital tract or intra-amniotic fluid. Late onset sepsis which occurs more than 72 hours after birth is acquired by pathogens in the environment [30]. Antibiotic use among neonates in selected hospitals has previously been described in different prospective cohort studies. For example, Zingg et al found that 26.7% of neonates in Geneva, Switzerland, were treated with antibiotics for an average of 6.8 days [31], while Cantey et al found that 72% of the neonates in Texas, US were treated with antibiotics for an average of 5.7 days [32]. However, neither of the studies addressed differences in antibiotic treatment between term and preterm neonates. Furthermore, due to differences in patient characteristics, level of health care and demographic factors it is difficult to compare results between single-center studies.

In our study, extremely preterm infants were twice as likely to receive antibiotic treatment during hospitalisation compared to term infants, while no significant difference could be seen between moderately preterm and term infants. As a group, extremely preterm infants have very high morbidity; half of surviving infants have been estimated to suffer from severe neonatal morbidities [33]. In contrast, moderately preterm infants have a health status more like term infants, which could explain the similar results between these moderately preterm and term infants.

Håkansson et al studied antibiotic use in neonatal wards on a national level, through the Swedish Neonatal Quality Register [30]. A strong correlation was seen between days of antibiotic therapy and gestational age; days of therapy varied between a mean of 1 day of therapy in term infants to 37 days of therapy in the extremely premature infants. In contrast, we did not find a significant difference in days of therapy between term, moderately preterm and extremely preterm individuals. This could be due to the wider range of age our study covered (first year of life compared to the neonatal period) and also that we included antibiotic treatment independent of diagnosis, while Håkansson et al focused mainly on infants with neonatal sepsis. We observed a difference in age distribution between term and preterm infants in our study population. This is likely due to the fact that the indications for hospitalisation differ between term and preterm, since a larger proportion of preterm infants are hospitalised in connection to birth compared to term infants. Since the difference in age between the two groups could affect our results, sub-analyses were performed for infants 0–3 months. However, the results did not differ compared to the entire study population.

The strength of our study is the initial cohort of women and infants identified from the Swedish nation-wide population-based health registers. Since our cohort included all hospital admissions for the selected diagnoses in Sweden between July 2005 and November 2011, the risk for selection bias is minimised. Furthermore, the response rate of medical records for both groups of women and infants was high, which is important for the results to be representative of the target population. By combining register data with patient data from medical records, we were able to study individual antibiotic treatment in relation to delivery mode and prematurity within hospitals, in a way not previously performed in Sweden.

The study has some limitations. Firstly, we had relatively small sample sizes, resulting in wide confidence intervals. Our results should therefore be interpreted with caution due to possible lack of power. Secondly, the reliability of information about antibiotics in the medical records could be discussed. Antibiotics were only used in 62% of emergency CS, which is low considering current guidelines. It is possible that antibiotics may have been given in reality, but that it was not stated in the discharge summary or included in the provided drug-record. Thirdly, the information regarding days of therapy was incomplete on several occasions. Our data included antibiotics given to the woman during the entire time of hospitalisation but due to lack of information on start and end date of antibiotic exposure, we cannot draw any conclusions regarding if the antibiotics was given peri-partum or post- partum in this group. Finally, since preterm infants are at a higher risk of exposure to intra-uterine infection due to chorioamnionitis (34) and pPROM (18) it would have been interesting also to study these conditions; however our sample size was too small.

In conclusion, women delivering by emergency CS had a higher probability of treatment with antibiotics than other modes of delivery and all CS had an increased length of stay compared to VD. These results confirm current guidelines. Extremely preterm infants were twice as likely to receive antibiotic treatment compared to term infants. Preterm infants overall were not more likely to receive antibiotic treatment, although a dose-response trend could be observed for degree of prematurity. The hospital stay was longer in preterm infants. Since exposure to antibiotics in early life may be linked to long term health consequences, difference in antibiotic treatment between the groups may be taken into consideration in future studies investigating the long term health effects of antibiotic exposure in early life.

## Supporting Information

S1 Table Risk ratios (RR) and 95% confidence intervals (CI) for antibiotic treatment by prematurity, sex and age in infants 0–3 months of age. (DOCX)
