# Supplementary material for: Antibiotic Treatment and Length of Hospital Stay in Relation to Delivery Mode and Prematurity
Source: PLoS One. 2016 Oct 7;11(10):e0164126. doi: 10.1371/journal.pone.0164126 (PMC5055307; doi:10.1371/journal.pone.0164126)
Supplement: S1 Table — (DOCX) [file pone.0164126.s001.docx]

**S1 Table. Risk ratios (RR) and 95% confidence intervals (CI) for antibiotic treatment by prematurity, sex and age in infants 0-3 months of age (N=389).**

|  |  | All | Antibiotics | |  |
| --- | --- | --- | --- | --- | --- |
|  |  | N | No, n (%) | Yes, n (%) | RR (95% CI) |
| **All** |  | **389** | **295 (75.8)** | **67 (17.2)** | **-** |
| **Prematurity** | Term | 142 | 112 (78.9) | 30 (21.1) | 1 (ref) |
|  | Preterm | 247 | 183 (74.1) | 64 (25.9) | 1.2 (0.8-1.8) |
|  | *Moderately* | 223 | 172 (77.1) | 51 (22.9) | 1.1 (0.7- 1.6) |
|  | *Extremely* | 24 | 11 (45.8) | 13 ( 54.2) | 2.6 (1.6-4.2) |
| **Sex** | Male | 224 | 171 (76.3) | 53 (23.7) | 1 (ref) |
|  | Female | 165 | 124 (75.2) | 41 (24.9) | 1.1 (0.7-1.5) |
